# A Meta‐Analysis of the Effects of Early Life Stress on the Prefrontal Cortex Transcriptome Reveals Long‐Term Downregulation of Myelin‐Related Gene Expression

**DOI:** 10.1002/brb3.70608

**Published:** 2025-06-17

**Authors:** Toni Q. Duan, Megan H. Hagenauer, Elizabeth I. Flandreau, Anne Bader, Duy Manh Nguyen, Pamela M. Maras, Randriely Merscher S. De Lima, Trevonn Gyles, Christabel Mclain, Michael J. Meaney, Eric J. Nestler, Stanley J. Watson, Huda Akil

**Affiliations:** ^1^ Grinnell College Grinnell Iowa USA; ^2^ Michigan Neuroscience Institute University of Michigan Ann Arbor Michigan USA; ^3^ Psychology Department Grand Valley State University Allendale Michigan USA; ^4^ McGill University Montreal Quebec Canada; ^5^ Nash Family Department of Neuroscience and Friedman Brain Institute Icahn School of Medicine at Mount Sinai New York City New York USA; ^6^ Douglas Hospital Research Centre Verdun Quebec Canada

**Keywords:** early life stress, meta‐analysis, microarray, RNA‐Seq

## Abstract

**Background:**

Early life stress (ELS) refers to exposure to negative childhood experiences, such as neglect, disaster, and physical, mental, or emotional abuse. ELS can permanently alter the brain, leading to cognitive impairment, increased sensitivity to future stressors, and mental health risks. The prefrontal cortex (PFC) is a key brain region implicated in the effects of ELS.

**Methods:**

To better understand the effects of ELS on the PFC, we ran a meta‐analysis of publicly available transcriptional profiling datasets. We identified five datasets (GSE89692, GSE116416, GSE14720, GSE153043, and GSE124387) that characterized the long‐term effects of multiday postnatal ELS paradigms (maternal separation, limited nesting/bedding) in male and female laboratory rodents (rats, mice). The outcome variable was gene expression in the PFC later in adulthood as measured by microarray or RNA‐Seq. To conduct the meta‐analysis, preprocessed gene expression data were extracted from the Gemma database. Following quality control, the final sample size was *n* = 89*(n* = 42 controls and *n* = 47 ELS: GSE116416, *n* = 23 (no outliers); GSE116416, *n* = 44 (two outliers); GSE14720, *n* = 7 (no outliers); GSE153043, *n* = 9 (one outlier); and GSE124387, *n* = 6 (no outliers)). Differential expression was calculated using the *limma* pipeline followed by an empirical Bayes correction. For each gene, a random‐effects meta‐analysis model was then fit to the ELS versus control effect sizes (Log2 Fold Changes) from each study.

**Results:**

Our meta‐analysis yielded stable estimates for 11,885 genes, identifying five genes with differential expression following ELS (false discovery rate < 0.05) — transforming growth factor alpha (*Tgfa*), IQ motif containing GTPase activating protein 3 (*Iqgap3*), collagen, type XI, alpha 1 (*Col11a1*), claudin 11 (*Cldn11*), and myelin‐associated glycoprotein (*Mag*) — all of which were downregulated. Broadly, gene sets associated with oligodendrocyte differentiation, myelination, and brain development were downregulated following ELS. In contrast, genes previously shown to be upregulated in major depressive disorder patients were upregulated following ELS.

**Conclusion:**

These findings suggest that ELS during critical periods of development may produce long‐term effects on the efficiency of transmission in the PFC and drive changes in gene expression similar to those underlying depression.

## Graphical abstract

To elucidate the long‐term effects of early life stress (ELS) on the prefrontal cortex (PFC), we conducted a meta‐analysis of five publicly available transcriptional profiling datasets from adult rodents that had experienced ELS. We observed consistent downregulation of myelin‐related genes following ELS and upregulation related to major depressive disorder.

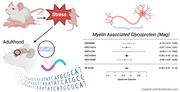

## 1 Introduction

Early life adversity, also known as early life stress (ELS), refers to exposure to negative childhood experiences. ELS can be caused by physical, mental, or emotional abuse, war, disaster, malnutrition, neglect, or negative parenting (Malave et al. 2022). Unfortunately, many children and adolescents around the world experience ELS, with one in six children facing four or more adverse childhood experiences (Malave et al. 2022). These adverse experiences can have a long‐lasting impact: ELS can permanently alter the brain (Barnett Burns et al. 2018), leading to cognitive impairment, increased stress sensitivity, conduct disorder, mental health risks, reduced antidepressant response, and other irreversible changes that persist into adulthood (Malave et al. 2022).

Researchers leverage animal models to study the effects of ELS on the brain. Laboratory‐reared rodents reach significant developmental milestones rapidly, progressing from birth to weaning in 3 weeks (Postnatal [P] Days P0–P21). The formation and functional maturation of the brain are fundamentally shaped during this early postnatal period (Short and Baram 2019). During this critical period, certain experiences can cause irreversible changes to neural circuitry (Short and Baram 2019). These periods of heightened plasticity are determined by neurobiological processes such as excitation and inhibition balance, synaptogenesis, synaptic pruning, myelination, and neurogenesis (Malave et al. 2022).

Much like humans, maternal care is critical for rodent development (Chen and Baram 2016). Therefore, maternal separation is a widely studied animal model of ELS (Plotsky and Meaney 1993). During a maternal separation experiment, dams are separated from pups daily for 1–8 h during the first 2–3 weeks of life. Maternal separation in rats increases offspring anxiety‐like behaviors long‐term. The effects of maternal separation on anxiety‐like behaviors are larger following a longer separation period (Plotsky and Meaney 1993; Malave et al. 2022), whereas a briefer separation, called early handling, can be protective (Pryce et al. 2005).

A newer rodent model of ELS is limited bedding and nesting material (Rice et al. 2008). In this model, rodent dams and their pups are placed on a wire mesh with inadequate nesting and bedding material for the first week of life. This model replicates impoverished housing conditions by preventing the mother from building a suitable nest. Limited bedding and nesting material cause maternal care to become unpredictable, producing recurrent threats and abusive behavior (Rice et al. 2008) and low pup body weight prior to weaning, suggesting malnutrition (Gilles et al. 1996; Malave et al. 2022; Rice et al. 2008).

In both rodents and humans, ELS affects many key brain regions, including the hippocampus, anterior cingulate, ventral tegmental area, and prefrontal cortex (PFC) (Luo et al. 2023; Malave et al. 2022; Peña et al. 2017, 2019). The PFC is a key region within the corticolimbic system, with the medial portion (mPFC) playing a central role in short‐term memory, working memory, decision‐making, and emotional regulation (McKlveen et al. 2019). Many developmental processes proceed in a caudal to rostral direction in the brain, with frontal, phylogenetically recent structures such as the PFC completing some processes, such as myelination, in late adolescence or early adulthood (human, ages 17–25 years; rat, Postnatal Day P90) (Zeiss 2021). Due to its prolonged developmental trajectory and high concentration of glucocorticoid receptors, the mPFC is especially vulnerable to the effects of ELS (McKlveen et al. 2019). Emotional and social brain networks centered on the PFC can be particularly affected by ELS, as can networks associated with executive functions and memory (Malave et al. 2022).

To better understand the effects of ELS on the brain, we conducted a meta‐analysis of publicly available transcriptional profiling datasets from rodent models. Transcriptional profiling technologies, such as RNA‐Seq or microarray, provide insight into gene activity and quantify expression levels in tissue samples, offering insights into biological processes, disease mechanisms, and treatment response. Despite the richness of this information, conclusions drawn from individual transcriptional profiling studies are often limited due to small sample sizes and technical noise. Meta‐analysis is a useful tool for addressing these issues. By compiling data from several studies, meta‐analysis can increase statistical power, identify consistent patterns across datasets, and uncover more robust biological patterns. This allows us to derive more reliable conclusions and discover novel associations that might not otherwise be apparent.

## 2 Materials and Methods

### 2.1 General

The meta‐analysis pipeline was adapted from the *Brain Data Alchemy Project* (protocol: M. Hagenauer et al. 2024, *not pre‐registered*) and associated code repository (https://github.com/hagenaue/BrainDataAlchemy). Analyses were conducted in R Studio using the language R (R v.4.2.1, R Studio v. 2023.06.0). All analysis code is available at https://github.com/tonid2/EarlyLifeStressSummer2023.

To perform our meta‐analysis of public transcriptional profiling data, we leveraged the data curation, preprocessing, and analysis efforts of the Gemma database (Lim et al. 2021). As of date, the Gemma project has reprocessed nearly 20,000 publicly available transcriptional profiling datasets. Gemma's preprocessing and data analysis steps adhere to a standardized pipeline. Gemma first realigns the datasets to an updated genome. Gemma performs quality control including identification and removal of outlier samples, removal of genes (rows) with minimal variance in expression values (either zero variance or < 70% distinct values), and manual curation for common issues such as batch effects (Lim et al. 2021). Differential expression is calculated using the limma or limma‐voom pipeline, and statistical output is available for the full model (omnibus) and individual contrasts.

### 2.2 Dataset Identification

**FIGURE 1:**
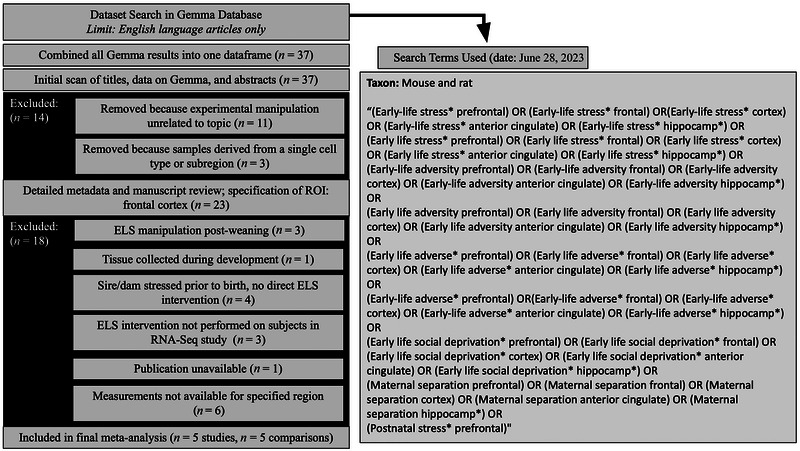
Diagram overviewing dataset search and selection. We identified transcriptional profiling datasets examining ELS models in laboratory rodents within the Gemma database using prespecified keywords. Our initial search encompassed tissue from three brain regions implicated in the effects of ELS: the hippocampus, anterior cingulate, and prefrontal cortex (PFC). The focus of our meta‐analysis was later narrowed to the PFC based on the availability of datasets. The titles, abstracts, and metadata for the datasets were initially scanned and filtered using prespecified inclusion/exclusion criteria, including indications that the dataset was not derived from a bulk dissection of brain tissue or that the content was unrelated to the research question (ELS). The secondary screening step included a detailed review of the metadata on Gemma and published methodology, followed by a final specification of the region of interest (ROI: PFC). Datasets were excluded in the secondary dataset filtering if they did not include an ELS manipulation during the preweaning period, the publication was not publicly available, tissue was collected during development (instead of adulthood), or transcriptional profiling was not performed for the ROI. *n*, number of datasets.

The *search_datasets()* function within the *gemma.R* package (gemma.R_1.3.2, https://github.com/PavlidisLab/gemma.R; Lim et al. 2021; Zoubarev et al. 2012) was used to find transcriptional profiling datasets from laboratory rats (*Rattus norvegicus*) or mice (*Mus musculus*) within the Gemma database containing prespecified keywords (Figure 1). We searched for datasets derived from three well‐studied brain tissues implicated in ELS (regions of interests or ROIs)—the hippocampus, anterior cingulate, and PFC (Luo et al. 2023; Malave et al. 2022; Peña et al. 2017, 2019)—with the intention of later narrowing the focus to a single brain region based on the number of available datasets to limit our research topic to what was feasible in the available time frame.

### 2.3 Dataset Filtering

The initial dataset filtering consisted of scanning dataset titles and metadata. Metadata records were filtered down to publicly available datasets, which were not labeled problematic within the Gemma database. Dataset titles, abstracts, and metadata were scanned for immediate indications that the content did not meet prespecified inclusion/exclusion criteria (protocol: M. Hagenauer et al. 2024) by a single researcher (T.Q.D.). Datasets were excluded if the experimental manipulation was unrelated to the meta‐analysis topic (rodent models of ELS). Datasets were also excluded if samples did not represent bulk brain tissue (e.g., scRNA‐Seq, laser capture microscopy, and cell sorting). A second researcher (M.H.H.) then approved all inclusion/exclusion choices.

The secondary dataset filtering involved specifying the brain ROI and then conducting detailed methodological reviews using both the available metadata and full‐text manuscripts. As a brain ROI was not fully prespecified in the search criteria, the research question was now narrowed based on dataset availability and filtered accordingly. For the three potential brain ROIs, five available datasets represented the PFC, five represented the hippocampus, and no datasets represented the anterior cingulate. The PFC was chosen as the final brain ROI.

The remaining datasets were then subjected to a detailed review of accompanying experimental methods and available file formats. Publications associated with the datasets were referenced in addition to detailed metadata on Gemma. Only methodological information from the publications was used to determine inclusion/exclusion. During this review, we used prespecified inclusion/exclusion criteria (M. Hagenauer et al. 2024) and criteria specific to our research question, including the requirement that the ELS occur during the postnatal preweaning period and tissue be collected in adulthood. One experiment was excluded due to a lack of accompanying publication and methodological information (GSE23728)—this exclusion could artificially inflate effect sizes in our results, as there is a well‐known bias against publishing negative results. Another experiment that was included in the meta‐analysis provided a prenatal immune challenge (lipopolysaccharide on Embryonic Day 17) in addition to ELS. Otherwise, the risk of systematic bias within the datasets from the individual transcriptional profiling studies was deemed low for any particular gene‐level result, as the original publications conducted full genome analyses. At this stage, all inclusion/exclusion decisions were reviewed by the entire 2023 cohort of the *Brain Data Alchemy Project* (M.H.H., E.I.F., A.B., T.G., and C.M.).

### 2.4 Overview of the Final Included Datasets

After initial and secondary dataset filtering, a total of five datasets fulfilled all requirements for use in the meta‐analysis (Table 1: GSE89692, GSE116416, GSE14720, GSE153043, GSE124387—*please note that GSE124387 was initially accidentally miscategorized as an exclusion*). Out of the five, three datasets were measured by RNA‐Seq, while two were measured by microarray. The ELS paradigms included some combination of maternal separation for > 3 h/day or limited nesting and bedding in male and female rodents (mice, rats). Rodents were stressed during preweaning, within time periods ranging from Postnatal Day 1 (P1) to Day 21 (P21). In all five studies, PFC tissue was collected later during adulthood; two studies specifically sampled the medial PFC.

**TABLE 1:** Overview of studies included in the meta‐analysis.

GEO ID number	*n* (full dataset)	Number of outliers	Final *n* (Ref)	Final *n* (ELS)	Species	Author	Year	Platform	Tissue	Type	Duration	Age ELS	Age tissue	Sex	Adult stress
**GSE89692**	163*	0	12	11	Mouse	Peña et al.	2017, 2019	Illumina (RNA‐Seq)	mPFC	Maternal separation and limited nesting	4 h/day separation	P10–P20 (male); P10–P17 (female)	Adult	Both	CSD (males), STVS (females), Behav. Tests
**GSE116416**	47	2	20	24	Mouse	Rincel et al.	2019	Agilent (microarray)	mPFC	Maternal separation, maternal stress, and prenatal LPS	3 h/day separation	P2–P14; prenatal LPS: E17	∼P165	Both	Behav. Tests, blood collection
**GSE14720**	11	0	3	4	Rat	Benekareddy et al.	2010	Agilent (microarray)	PFC	Maternal separation	3 h/day separation	P2–P14	P60–P90	Male	Injection
**GSE153043**	10	1	4	5	Rat	Green et al.	2021	Illumina (RNA‐Seq)	PFC	Limited nesting	Full day	P2–P9	P91	Male	Behav. Tests
**GSE124387**	9	0	3	3	Rat	Zheng et al.	2019	Illumina (RNA‐Seq)	PFC	Maternal separation	4 h/day separation	P1–P21	After P96	Male	Anesthesia, Behav. Tests

*Note*: Within the table, “GEO ID number” refers to the Gene Expression Omnibus (GEO) accession number for the dataset. The sample size (*n*) for the full dataset is provided, as well as the final sample size for the control (Ref) and ELS groups included in the subset of the dataset used for our analysis (final total *n*: Ref = 42, ELS = 47). The subsetted sample size only includes the ROI (prefrontal cortex [PFC] or medial PFC [mPFC]) and excludes outlier samples. *Please note that each sample in GSE89692 represents RNA pooled from three subjects. The “Author” and “Year” refer to the publication (Benekareddy et al. 2010; Oldham Green et al. 2021; Peña et al. 2017; Rincel et al. 2019; Zheng et al. 2019). “Duration” indicates how long (hours: “h”) the pups experienced maternal separation each day; limited nesting and bedding lasted all day. The “P” under “Age ELS” represents the postnatal days during which the rodents received ELS. In GSE116416, the ELS group also received a prenatal immune challenge of lipopolysaccharide (LPS) on Embryonic Day 17 (E17). “Age tissue” indicates the age of sacrifice, when the tissue from the ROI was collected. “Adult stress” describes any additional stress exposure in adulthood, including chronic social defeat (CSD) in males, social threat vocalization stress (STVS) in females, behavioral tests (Behav. Tests), blood collection, injections, or anesthesia, as specified for each study. All experimental details refer specifically to the samples used in the transcriptional profiling experiment and not the full paper.

### 2.5 Individual Dataset Reprocessing: Outliers, Gene Filtering, and Batch Effects (Quality Control)

When possible, the preprocessed gene expression data for the selected experiments were reanalyzed to double‐check data quality and ensure preferred sample subsetting and differential expression model specification (GSE89692, GSE116416, GSE14720, and GSE153043). To do this reanalysis, summarized experiment objects for each study were read into R using *Gemma.R* to access the API for the Gemma database (July 2023). For GSE124387, the preprocessed gene expression data (summarized experiment object) were not available. For this dataset, we reviewed the data quality and model specification provided by Gemma and have provided that information below.

Each of the summarized experiment objects was subsetted to the relevant samples for the meta‐analysis using “organism part” (ROI: PFC) and “treatment” (only ELS and reference subject role). Gemma defines outliers as samples with an adjusted median sample–sample correlation outside the interquartile range of the sample–sample correlations for the sample with the closest median correlation to them (Lim et al. 2021). We double‐checked whether individual samples, as well as pairs or groups of outliers, met similar criteria regarding distance from the rest of the dataset by visualizing the distribution of sample–sample correlations within each dataset (correlation matrix, boxplots). After visualization, one outlier was removed from GSE153043, while two outliers were removed from GSE116416 (Table 1; Figure S1).

Within the Gemma database, batch‐related variables are combined into a single column (block); we re‐separated them using *strsplit()*. For RNA‐Seq, these variables included scan date, device, and run, which often impact gene expression measurements, as well as lane and flow cell. To determine if batch‐related variables might confound our design, we created cross‐tables comparing each batch‐related variable to our main variables of interest. We also examined the relationship between library size and our variables of interest using boxplots. To determine the impact of batch‐related variables, we used principal components analysis (PCA) to identify the main patterns of variation within gene expression data (function *prcomp(x*, *scale = TRUE)* in package: *stats*) on the transposed log2 gene expression matrix. The top four principal components of variation were compared to our variables of interest and potential nuisance variables (batch, library size, and other experimental factors in the dataset) using boxplots and scatterplots. This information guided decisions regarding the inclusion of covariates in the differential expression model for each dataset (Figure S1).

Only one dataset ended up having a differential expression model that included covariates (GSE89692; Equation 1). The differential expression models for the other datasets were kept simple (Equation 2), as these datasets were either very small (GSE153043, GSE14720, and GSE124387) or processed in a single batch (GSE116416), and contained samples with homogenous features within the specified subset.

*The differential expression model for GSE89692*:

(1) y∼Intercept+Treatment_factor+LibrarySize+Sex_factor

*The differential expression model for GSE116416*, *GSE153043*, *GSE14720*, *and GSE124387*:

(2) y∼Intercept+Treatment_factor

To mirror Gemma's pipeline, we calculated ELS versus control differential expression using the *limma* pipeline, accounting for the mean–variance relationship present in the datasets (*limma‐trend*, GSE124387: *limma‐voom*), and then applied an empirical Bayes correction to the differential expression results using the *eBayes()* function (Ritchie et al. 2015).

### 2.6 Result Extraction

The result extraction process followed the *Brain Data Alchemy* Pipeline (2023: M. Hagenauer et al. 2024). Using either our own reprocessed differential expression results (GSE89692, GSE116416, GSE14720, and GSE153043) or the differential expression results provided by Gemma (GSE124387), we extracted the Log2 Fold Changes (Log2FC) and sampling variances from the ELS versus control differential expression results for each dataset. To be included in the meta‐analysis, a gene needed to be present in the results from a minimum of four of the five datasets. Note that 11,889 genes fulfilled this criterion, and 11,885 genes produced stable meta‐analysis estimates using a simple (intercept‐only) random effects meta‐analysis model (*metafor* package: Viechtbauer 2010). The final sample size was not deemed large enough to conduct additional analyses to assess dataset heterogeneity or the effects of other potential variables of influence (e.g., ELS type). The meta‐analysis *p*‐values underwent false discovery rate (FDR) correction using the Benjamini–Hochberg method, also known as “*q*‐value” (function *mt.rawp2adjp(x, proc = c(“BH”))* in *multtest* package (v.2.8.0) (Pollard et al. 2005). Statistical significance was thresholded at 5% FDR (FDR < 0.05). To highlight key findings, forest plots depicting effect sizes (Log2FC) and their 95% confidence intervals from each contributing study were generated (function *forest.rma()* in package *metafor*).

### 2.7 Exploring Functional Patterns Within the Meta‐Analysis Results

*StringDB*: StringDB was used to explore potential protein–protein interactions (PPIs) between the top differentially expressed genes identified by the meta‐analysis. The StringDB database contains predicted protein–protein interaction information from numerous sources, including experimental data, computational predictions, and public text mining (https://string‐db.org/, accessed on September 27, 2024 [Szklarczyk et al. 2023]). Mouse gene symbols for the top genes (*p* < 0.001: 40 genes) from our meta‐analysis were entered into StringDB (minimum required interaction score: medium confidence (0.40), species: *Mus musculus)*. When examining the functional gene sets enriched within our network, we used a statistical background of all 11,889 genes included in our meta‐analysis.

*Brain.GMT*: Brain.GMT is a curated database of 918 gene sets related to nervous system function, tissue, and cell types. A version of Brain.GMT that has been combined with a traditional gene ontology gene set database (MSigDB “C5”: 14,996 gene sets) can be used within common analysis pipelines to improve the interpretation of neuroscience differential expression results (M. H. Hagenauer et al. 2024). We adapted the example R code accompanying the release of Brain.GMT for use within fast gene set enrichment analysis (fGSEA) (Korotkevich et al. 2021). Prior to running the code, gene sets were trimmed out of the.GMT file that were specifically derived from non‐cortical brain tissues (hippocampus or nucleus accumbens, as marked in Table 1 of the Brain.GMT paper: M. H. Hagenauer et al. 2024) or cell types (as marked “C8” in Table 1 of the Brain.GMT paper: M. Hagenauer et al. 2024), with the exception of bone marrow, which can reflect the blood cell types found in brain tissue.

## 3 Results

**FIGURE 2:**
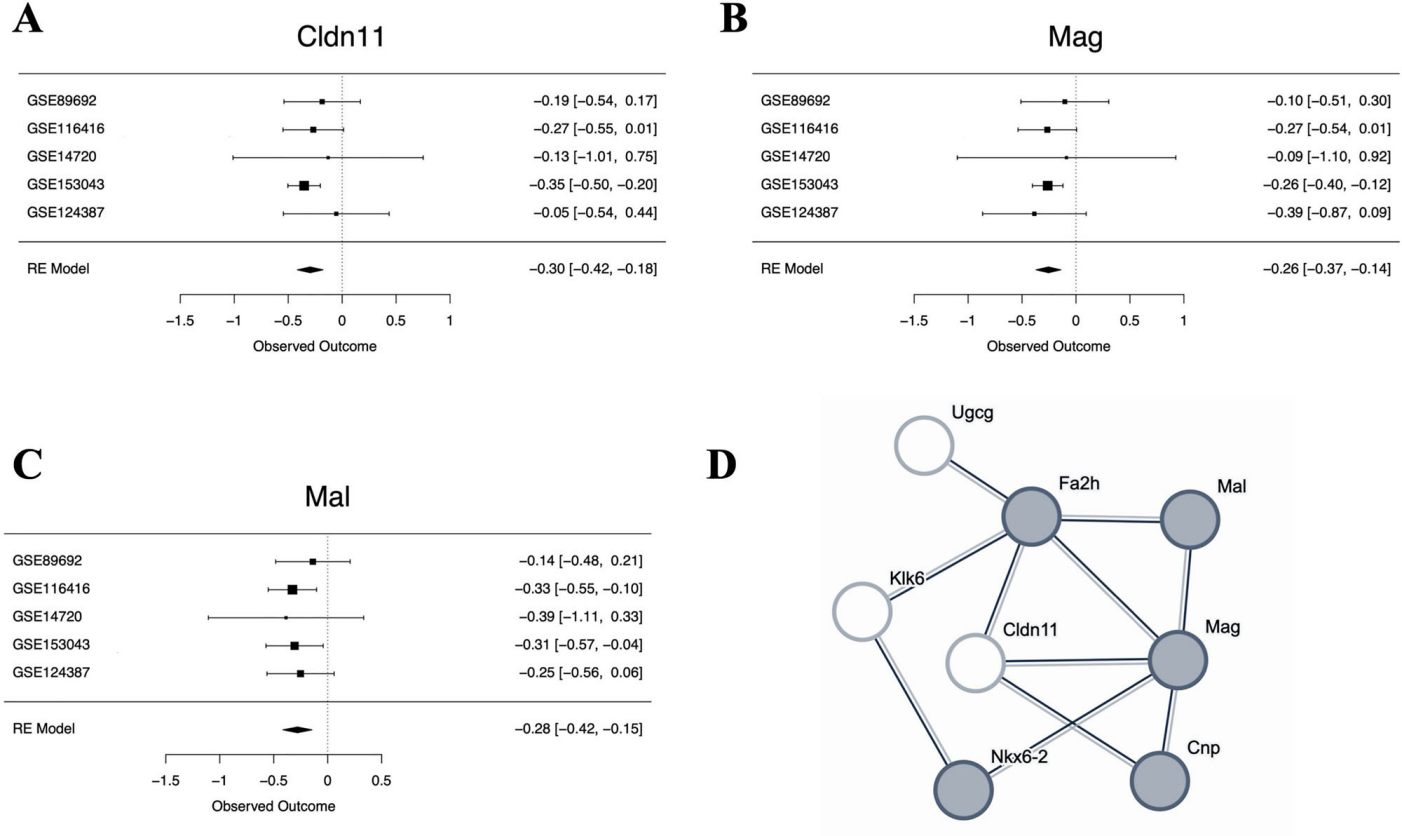
Genes within a network focused on oligodendrocyte differentiation are differentially expressed in early life stress models. (A–C) Forest plots for two differentially expressed genes from the meta‐analysis (FDR < 0.05: *Cldn11* and *Mag*) and a third gene that shows a trend toward differential expression (FDR < 0.10: *Mal*) that are all part of a network related to oligodendrocyte differentiation. Rows illustrate ELS Log2FC (squares) with 95% confidence intervals (whiskers) for each of the datasets and the meta‐analysis random effects model (RE Model). Forest plots allow for visual inspection of the consistency and magnitude of effects across the five studies. (A) A forest plot showing the downregulation of claudin 11 (*Cldn11*) in ELS models. (B) A forest plot showing the downregulation of myelin‐associated glycoprotein (*Mag*) in ELS models. (C) A forest plot showing the trend toward a downregulation of mal, T cell differentiation protein (*Mal*) in early life stress models. (D) Multiple top differentially expressed genes are found in the same predicted protein–protein interaction (PPI) network enriched for genes associated with oligodendrocyte differentiation. To create the PPI network, top genes from the meta‐analysis (*p* < 0.001: 40 genes) were entered into the StringDB database. The only identifiable network (cluster) of eight genes included six genes with either significant differential expression in ELS models (FDR < 0.05: *Mag* and *Cldn11*) or a nonsignificant trend toward differential expression in ELS models (FDR < 0.10: *Mal, Fa2h, Klk6*, and *Ugcg*). The nodes represent proteins and are labeled with mouse gene symbol annotation. The lines represent predicted protein–protein associations. The associations are meant to be specific and meaningful (proteins jointly contribute to a shared function), but this does not necessarily mean they are physically binding to each other. The shaded nodes (gray) were identified as being part of a gene set involved in oligodendrocyte differentiation (GO:0048709) that was enriched with differential expression. Other genes within the network are part of a gene set involved in central nervous system myelination (GO:0022010) that was also enriched with differential expression (including *Klk6*) or have also been previously associated with oligodendrocyte function and myelination (*Cldn11*).

To be included in the meta‐analysis, a gene needed to be present in the results from a minimum of four out of five datasets. Note that 11,889 genes fulfilled this criterion, and 11,885 genes produced stable meta‐analysis estimates (**Table** **S1**). Of the 11,885 genes represented in the meta‐analysis, five genes were significantly differentially expressed (FDR < 0.05) in ELS models (Table 2): transforming growth factor alpha (*Tgfa*; Figure S2A), claudin 11 (*Cldn11*; Figure 2A), myelin‐associated glycoprotein (*Mag*; Figure 2B), collagen, type XI, alpha 1 (*Col11a1*; Figure S2B), and IQ motif containing GTPase activating protein 3 (*Iqgap3*; Figure S2C). All were downregulated. Twelve other genes showed a trend toward differential expression (FDR < 0.10), with six of those genes downregulated by ELS and six upregulated by ELS (Table 2).

**TABLE 2:** Meta‐analysis results: top differentially expressed genes (FDR < 0.10) in the PFC of adult rodents that had experienced ELS.

Rat Entrez gene ID	Mouse Entrez gene ID	Gene symbol	Log2FC estimate	SE	CI_lb	CI_ub	*p*‐value	Number of comparisons	FDR
293711	66727	*Plaat5*	−0.62	0.15	−0.92	−0.33	3.41E‐05	4	0.05543
**310621**	**404710**	***Iqgap3***	**−0.54**	**0.12**	**−0.77**	**−0.30**	**5.38E‐06**	**4**	**0.02133**
**25654**	**12814**	***Col11a1***	**−0.48**	**0.11**	**−0.70**	**−0.26**	**2.10E‐05**	**4**	**0.04998**
29245	19144	*Klk6*	−0.45	0.11	−0.67	−0.24	4.66E‐05	4	0.05543
**24827**	**21802**	***Tgfa***	**−0.35**	**0.06**	**−0.47**	**−0.22**	**9.60E‐08**	**4**	**0.00114**
307855	338521	*Fa2h*	−0.30	0.08	−0.45	−0.15	9.18E‐05	4	0.08395
**84588**	**18417**	***Cldn11***	**−0.30**	**0.06**	**−0.42**	**−0.18**	**1.10E‐06**	**5**	**0.00653**
114004	68458	*Ppp1r14a*	−0.29	0.08	−0.44	−0.14	1.31E‐04	5	0.09155
680723	320587	*Tmem88b*	−0.29	0.07	−0.43	−0.14	1.20E‐04	4	0.09155
25263	17153	*Mal*	−0.28	0.07	−0.42	−0.15	4.60E‐05	5	0.05543
**29409**	**17136**	***Mag***	**−0.26**	**0.06**	**−0.37**	**−0.14**	**1.56E‐05**	**5**	**0.04639**
83626	22234	*Ugcg*	0.09	0.02	0.04	0.13	8.62E‐05	5	0.08395
302032	232566	*Amn1*	0.09	0.02	0.04	0.14	1.28E‐04	4	0.09155
308718	18634	*Pex7*	0.09	0.02	0.05	0.14	9.12E‐05	5	0.08395
361006	76670	*Cfap70*	0.23	0.06	0.11	0.35	1.30E‐04	4	0.09155
25253	13482	*Dpp4*	0.33	0.08	0.18	0.49	3.23E‐05	4	0.05543
100363095	66715	*Henmt1*	0.40	0.10	0.21	0.59	4.26E‐05	4	0.05543

*Note*: Rows highlighted with bold text denote genes that are significantly differentially expressed (FDR < 0.05); all other genes show a trend toward differential expression (FDR < 0.10). The rows are ordered by Log2FC (lowest to highest). A positive Log2FC denotes that a gene's expression is higher in the experimental group compared to the control group (upregulated: highlighted gray), with higher positive values indicating greater increases. A negative Log2FC indicates lower expression in the experimental group relative to the control (downregulated), where larger negative values signify more substantial decreases. The number of comparisons represents the number of datasets that included measurements for the gene following quality control.

Abbreviations: CI_lb, confidence interval lower bound; CI_ub, confidence interval upper bound; FDR, false discovery rate (*q*‐value); Log2FC, Log2 Fold Change (ELS vs. control); SE, standard error.

To explore the biological functions associated with our differentially expressed genes, we first created a predicted PPI network by entering top genes from the meta‐analysis (*p* < 0.001: 40 genes) into the StringDB database. A network (cluster) of eight genes was identified that included several significantly differentially expressed genes (FDR < 0.05: *Cldn11* and *Mag*; Figure 2D). To identify functional gene sets enriched within our PPI network, we compared the top genes (*p* < 0.001: 40 genes) from the meta‐analysis to a statistical background of all 11,889 genes included in the meta‐analysis. Five of the genes present in the PPI network were identified as involved in *oligodendrocyte differentiation* (*GO:0048709*). In contrast, only 85 proteins in total (in the network and background) have this term assigned, indicating a 1.24× enrichment of overlap with our ELS network (FDR = 0.0488). Four genes within the PPI network were also associated with the related concept of *central nervous system myelination* (*GO:0022010*), out of the 27 proteins in total assigned this term (in the network and background), indicating a 1.64x enrichment of overlap with our ELS network (FDR = 0.0398).

For a more comprehensive identification of functional gene sets enriched with the effects of ELS in our meta‐analysis, we performed an fGSEA using a database containing both brain‐related functional gene sets (*Brain.GMT*) and traditional gene ontology gene sets. Of the 9322 gene sets included in the results, 42 had an enrichment of ELS effects surviving FDR correction (FDR < 0.05; Table S2). Of these 42 gene sets, 21 were from *Brain.GMT* and 21 were traditional gene ontology gene sets. Most of these enriched gene sets were downregulated following ELS (40 of 42) and explicitly related to oligodendrocyte development or myelination (22 of 42) or more broadly related to brain development (6 of 42). There were two notable exceptions: one downregulated gene set was related to stress hormone signaling (*GOBP_RESPONSE_TO_MINERALOCORTICOID*, strongest leading gene: neuronal PAS domain protein 4 (*Npas4*), Log2FC = −0.34, *p* = 0.012, FDR = 0.61), and one of the only upregulated gene sets was derived from a meta‐analysis characterizing the effects of major depressive disorder in the frontal cortex (*Gandal_2018_MajorDepressiveDisorder_Upregulated_Cortex*, strongest leading gene: follistatin (*Fst*): Log2FC = 0.14, *p* = 0.0092, FDR = 0.60). Twenty‐five of the enriched gene sets contained leading genes that were significantly differentially expressed genes following ELS in the meta‐analysis (*Tgfa*, *Cldn11*, *Mag*, and *Col11a1*; Table 3).

**TABLE 3:** Functional gene sets that are enriched with the effects of early life stress are often related to oligodendrocyte development and myelination.

Gene set	*p*‐value	FDR	ES	NES	Size	Leading genes
GOMF_SIGNALING_RECEPTOR_BINDING	0.00015	0.045	−0.31	−1.38	959	*Tgfa*, *Mag*
GOBP_BIOLOGICAL_ADHESION	0.00015	0.045	−0.31	−1.39	967	*Cldn11*, *Mag*
GOBERT_OLIGODENDROCYTE_DIFFERENTIATION_DN	0.00015	0.045	−0.37	−1.64	891	*Col11a1*
GOBP_NEURON_DEVELOPMENT	0.00015	0.045	−0.33	−1.47	892	*Mag*
HP_ONSET	0.00016	0.045	−0.32	−1.42	846	*Mag*
MEISSNER_BRAIN_HCP_WITH_H3K4ME3_AND_H3K27ME3	0.00016	0.045	−0.41	−1.77	829	*Cldn11*
GOBP_CENTRAL_NERVOUS_SYSTEM_DEVELOPMENT	0.00016	0.045	−0.41	−1.77	758	*Mag*
FAN_EMBRYONIC_CTX_OLIG	0.00016	0.045	−0.51	−2.19	614	*Cldn11*, *Mag*
GOBP_NERVOUS_SYSTEM_PROCESS	0.00016	0.045	−0.34	−1.46	655	*Mag*, *Col11a1*
HP_CONSTITUTIONAL_SYMPTOM	0.00016	0.045	−0.34	−1.46	643	*Col11a1*
Oligodendrocyte_All_Zeisel_Science_2015	0.00017	0.045	−0.59	−2.40	320	*Cldn11*, *Mag*
GOBP_GLIOGENESIS	0.00017	0.045	−0.51	−1.99	218	*Mag*
GOBP_GLIAL_CELL_DIFFERENTIATION	0.00018	0.045	−0.55	−2.09	168	*Mag*
DESCARTES_MAIN_FETAL_SCHWANN_CELLS	0.00018	0.045	−0.56	−2.10	159	*Mag*
ZHONG_PFC_C4_PTGDS_POS_OPC	0.00018	0.045	−0.52	−1.88	120	*Cldn11*, *Mag*
GOBP_ENSHEATHMENT_OF_NEURONS	0.00018	0.045	−0.60	−2.12	105	*Cldn11*, *Mag*
GOBP_GLIAL_CELL_DEVELOPMENT	0.00019	0.045	−0.60	−2.05	89	*Mag*
GOBP_OLIGODENDROCYTE_DIFFERENTIATION	0.00019	0.045	−0.63	−2.10	78	*Mag*
LEIN_OLIGODENDROCYTE_MARKERS	0.00019	0.045	−0.82	−2.69	70	*Mag*
DESCARTES_MAIN_FETAL_OLIGODENDROCYTES	0.00019	0.045	−0.69	−2.19	59	*Cldn11*, *Mag*, *Col11a1*
GOCC_MYELIN_SHEATH	0.00019	0.045	−0.75	−2.17	35	*Mag*
Oligodendrocyte_Myelinating_Zhang_JNeuro_2014	0.00019	0.045	−0.85	−2.47	35	*Cldn11*
Oligodendrocyte_All_Cahoy_JNeuro_2008	0.00019	0.045	−0.87	−2.48	33	*Cldn11*, *Mag*
DESCARTES_FETAL_CEREBRUM_OLIGODENDROCYTES	0.00019	0.045	−0.76	−2.18	33	*Cldn11*, *Mag*
Oligodendrocyte_Mature_Darmanis_PNAS_2015	0.00020	0.045	−0.91	−2.25	17	*Cldn11*, *Mag*

*Note*: Out of 42 gene sets that were enriched with the effects of ELS (FDR < 0.05) within a gene set enrichment analysis performed using the meta‐analysis results, most were downregulated following ELS (40 of 42) and explicitly related to oligodendrocyte development or myelination (22 of 42). Listed above are the 25 enriched gene sets that contained leading genes that were significantly differentially expressed following ELS in the meta‐analysis (*Tgfa*, *Cldn11*, *Mag*, and *Col11a1*). Size: size of gene set. Leading genes: A list of the leading genes in the gene set that were significantly differentially expressed following ELS (FDR < 0.05) in the meta‐analysis.

Abbreviations: ES, enrichment score; FDR, false discovery rate; NES, normalized enrichment score.

## 4 Discussion

Our research provides novel insight into the effect of ELS on the PFC. ELS is known to cause a profound and lasting impact on the brain, leading to cognitive impairments, heightened stress sensitivity, and increased mental health risk. Studying the impact of ELS on the PFC is important because the PFC plays a key role in cognitive functions like decision‐making, impulse control, emotional regulation, and social behavior (Arnsten 2009). During childhood and adolescence, the PFC undergoes significant development and experiences increased vulnerability to stress (Chocyk et al. 2013). When stress occurs during this critical period, it can alter prefrontal neural circuit development, heightening later risk for mental health disorders such as depression, anxiety, and PTSD (Colich et al. 2017). Additionally, ELS affects neuroplasticity—the brain's ability to adapt and reorganize—resulting in impairments in learning, memory, and executive function (Malave et al. 2022).

To gain deeper insights into how ELS influences the PFC, we conducted a meta‐analysis of publicly available transcriptional profiling datasets. We identified five relevant datasets (GSE89692, GSE116416, GSE14720, GSE153043, and GSE124387) that examined the long‐term effects of multiday ELS paradigms, such as maternal separation for >3 h per day and/or limited nesting material, on male and female laboratory pups (rats, mice) during the postnatal period (between P1–P21). The outcome variable was gene expression in the PFC in adulthood, as measured by microarray or RNA‐Seq. We achieved stable meta‐analysis estimates for 11,885 genes, identifying five key results that withstood FDR correction (FDR < 0.05): downregulation of *Tgfa*, *Cldn11*, *Iqgap3*, *Col11a1*, and *Mag*.

Notably, the proteins encoded by *Mag* and *Cldn11* are both important for myelination. *Mag* encodes a membrane protein within the immunoglobulin superfamily that is predicted to contribute to the maintenance of normal axon myelination and neuron–myelin communication (Quarles 2007). *Cldn11* produces a protein in the Claudin family, which is a crucial component of the tight junctions in cell membranes that prevent the free passage of substances between cells, maintaining cell structure and communication. Claudin 11 is a myelin component and an important regulator of oligodendrocyte proliferation and migration (Riedhammer et al. 2021). *Tgfa* was also downregulated and plays a protective role in maintaining oligodendrocyte viability and white matter integrity (Dai et al. 2020).

When conducting a gene set enrichment analysis to explore the functional patterns within our results, we identified 42 gene sets that were enriched with ELS effects, most of which were downregulated and related to brain development, especially oligodendrocyte development and myelination, led by downregulation of *Cldn11*, *Mag*, and *Col11a1*. We also observed an enrichment of upregulation of genes previously found to be upregulated in the cerebral cortex of patients with major depressive disorder. However, the strongest upregulated gene in this set, *Fst* —encoding an inhibitor of follicle‐stimulating hormone release—did not surpass our threshold for differential expression (FDR > 0.10) (Shi et al. 2016). The StringDB database similarly revealed that the proteins for downregulated genes *Cldn11* and *Mag* are part of a predicted PPI network with the proteins for other genes that trended toward significant differential expression (FDR < 0.10) in association with ELS in our meta‐analysis, including downregulated mal, T cell differentiation protein (*Mal*, also known as myelin and lymphocyte protein), kallikrein‐related peptidase 6 (*Klk6)*, and fatty acid 2‐hydroxylase *(Fa2h)*, and upregulated UDP‐glucose ceramide glucosyltransferase (*Ugcg)* (Szklarczyk et al. 2023). According to StringDB, this network is enriched with genes associated with oligodendrocyte differentiation and myelination, including *Mag*, *Mal*, *Klk6*, *Fa2h*, and *Ugcg*. Collectively, these findings suggest that ELS during critical developmental periods may lead to long‐term disruptions in myelin‐related processes, potentially affecting the efficiency of neural transmission in the PFC.

Altogether, our meta‐analysis supports previous evidence in rodents, demonstrating that ELS disrupts the normal development and differentiation of oligodendrocytes and the process of myelination, essential components of cortical white matter. For instance, separating rat pups from their mothers for 3 h daily during the first 3 weeks of life reduces myelination in the PFC (Yang et al. 2017). This reduction in myelination was linked to a decrease in mature oligodendrocytes and an increase in oligodendrocyte precursor cells (OPCs), suggesting that ELS impairs normal maturation, preventing OPCs from properly differentiating into mature myelinating cells. These myelination deficits were observed both after weaning (P21) and in adults (P60). Likewise, when mice were exposed to 3 h of split litter maternal separation (slMS) during the first 2 weeks of life, it caused early maturation of OPCs in the PFC by day 21 (P21), followed by their depletion in adulthood (Teissier et al. 2020). Isolating juvenile mice after weaning (P21–P35) similarly led to abnormal maturation of oligodendrocytes in the PFC and impaired performance on cognitive tasks (Makinodan et al. 2012). Of note, exposure of adult rodents to chronic social stress has also been shown to impair myelination in the PFC (Bonnefil et al. 2019; Liu et al. 2012).

Preventing disruptions in oligodendrocyte maturation can mitigate some long‐term behavioral effects of ELS. For example, maternal separation can cause a long‐term increase in WNT signaling, which blocks OPC differentiation and new myelination in the adult PFC (Yang et al. 2017). Administering a WNT antagonist (XAV939) during the first 3 weeks of life partially rescued the myelination and behavioral deficits observed after ELS. Likewise, inhibiting neuronal activity during the first weeks of life can lead to early myelination (Teissier et al. 2020) and behavioral problems resembling mice exposed to maternal separation (Teissier et al. 2020; Menezes et al. 2024), whereas activating PFC neurons during early postnatal weeks resulted in reduced myelination at P15 and increased myelination in adulthood (Teissier et al. 2020) and reversed the behavioral deficits observed in these models (Teissier et al. 2020; Menezes et al. 2024). These results make intuitive sense: since maternal care is a major source of environmental stimulation for young pups, it is unsurprising that maternal separation can disturb cortical activity (Sarro et al. 2014; Courtiol et al. 2018) and its associated activity‐dependent oligodendrocyte development (Nishiyama et al. 2021).

ELS has been linked to altered oligodendrocyte development and myelination in children, showing that these changes extend beyond animal models to human development. In functional imaging studies, children who have experienced ELS—such as early neglect, social deprivation, childhood maltreatment, or trauma—display long‐term disruptions to white matter. These effects include decreased white matter volume and microstructural abnormalities within tracts connecting the frontal lobes to other brain regions (Hanson et al. 2013; Govindan et al. 2010; Meinert et al. 2019). White matter microstructural abnormalities predict poorer neurocognitive performance (Hanson et al. 2013), suggesting that ELS‐induced abnormalities in white matter, particularly in the PFC, may contribute to resulting cognitive deficits (Hanson et al. 2013). Long‐term disruptions to white matter following ELS are also evident in postmortem tissue. Immunohistological studies performed on the ventromedial PFC indicate that a history of child abuse correlates with increased numbers of mature myelinating oligodendrocytes and decreased numbers of immature oligodendrocytes (Tanti et al. 2018). Within the nearby anterior cingulate, a history of child abuse correlates with a significant reduction in the thickness of myelin sheaths around small‐diameter axons and a global impairment of the myelin‐related transcriptional program (Lutz et al. 2017).

Our meta‐analysis both supports and extends these previous findings, offering a glimpse at the long‐term molecular changes in the PFC following ELS that may underlie myelination deficits. By using a meta‐analysis to study the effect of ELS on the PFC, we combined data from multiple studies to effectively reach a final sample size of *n* = 89, increasing statistical power to a level capable of detecting moderate effect sizes and providing more reliable effect estimates. Furthermore, by including studies from multiple ELS models (maternal separation, limited bedding and nesting) and laboratory settings in our meta‐analysis, we identified consistent patterns and enhanced the generalizability of our findings. However, we did not have the power to systematically evaluate sources of heterogeneity within the results, as only five final studies survived our inclusion/exclusion criteria for the meta‐analysis. Potential sources of heterogeneity include variables previously demonstrated to impact the influence of ELS on both the brain and behavior, including sex (Breton et al. 2021; Goodwill et al. 2019; Peña et al. 2019; Orso et al. 2025), species (Wang et al. 2020), developmental timing (Abraham et al. 2023), ELS model type (de Lima et al. 2020), and the presence of additional stress in adulthood (Kuhlman 2024). Our datasets were also skewed toward the use of males, mice, and maternal separation, raising questions about the generalizability of our results. That said, we did not observe notable heterogeneity in the differential expression of the myelin‐related genes in relationship to the ELS model type or the other aforementioned variables, as—by definition—the top results in our small meta‐analysis were elevated by consistency across datasets.

Our statistical power was limited by our high standards for the included studies—for example, we rejected one dataset that lacked an associated publication (GSE23728), preventing us from fully reviewing its methodology. To maintain consistency in data processing, we also only extracted preprocessed transcriptional profiling datasets from the Gemma database, precluding the use of data released on less standardized platforms (e.g., Github: Orso et al. 2025). As future datasets measuring the effects of ELS on the PFC are released, updating our meta‐analysis will likely result in additional significant findings. Likewise, previous data suggest that the effects of ELS on oligodendrocyte differentiation and myelination are likely to be present in other brain regions (Abraham et al. 2023; Govindan et al. 2010; Meinert et al. 2019; Lutz et al. 2017; Breton et al. 2021), with varying effects depending on the timing of ELS exposure in relation to regional brain development (Abraham et al. 2023). Therefore, future meta‐analyses examining the effects of ELS may be enriched by the inclusion of data from other well‐studied brain regions (e.g., the hippocampus: Alberry and Singh 2020; Bolton et al. 2020; Kiser et al. 2019; Peña et al. 2017; Suri et al. 2014) or developmental time frames (e.g., postweaning: Rawat et al. 2022; Sharma et al. 2023; Usui et al. 2021; Zhang et al. 2022). The question of how the developmental timing of ELS affects brain maturation may be of particular interest, as different stages of oligodendrocyte development occur in waves. The ELS interventions included in our meta‐analysis overlapped the developmental peak in oligodendrocyte proliferation (P0–P7) and oligodendrocyte differentiation (P14–P21), but only partially overlapped peak myelin development (P14–P30) (Nishiyama et al. 2021). Previous studies suggest that ELS during postweaning (P21–P35) in mice may also disrupt critical oligodendrocyte development and myelination in the mPFC (Makinodan et al. 2012), which is crucial for behavioral control and cognitive function in adulthood (Giedd 2004; McDougall et al. 2018; Mengler et al. 2014).

## 5 Conclusion

Approximately 65% of people in the United States experienced at least one type of ELS, while 12.5% experienced up to four (Fogelman and Canli 2019). Characterizing the impact of ELS on the PFC is crucial for understanding the long‐term cognitive and behavioral effects. Our results suggest that ELS during critical periods of development may produce long‐term effects on molecular contributors to oligodendrocyte development and myelination, potentially altering the efficiency of transmission in the PFC. Impaired PFC connectivity could reduce cognitive control and emotional regulation (Friedman and Robbins 2022) and disinhibit stress‐ and fear‐related neural circuits, increasing susceptibility to psychiatric disorders (Battaglia et al. 2024; Mayberg et al. 1999; Kessler et al. 2020). Our results also indicate that ELS can drive gene expression changes similar to those observed in the frontal cortex of patients with major depressive disorder. This knowledge is vital for developing targeted interventions to reduce the negative consequences of ELS and improve mental health outcomes.

## Author Contributions

**Toni Q. Duan**: conceptualization, methodology, software, data curation, investigation, formal analysis, visualization, writing–original draft, writing–review and editing. **Megan H. Hagenauer**: conceptualization, methodology, writing–review and editing, visualization, supervision, project administration, software, formal analysis, investigation. **Elizabeth I. Flandreau**: methodology, writing–review and editing. **Anne Bader**: methodology, writing–review and editing. **Duy Manh Nguyen**: methodology, writing–review and editing. **Pamela M. Maras**: methodology, writing–review and editing. **Randriely Merscher S. De Lima**: methodology, writing–review and editing. **Trevonn Gyles**: methodology, writing–review and editing. **Christabel Mclain**: methodology, writing–review and editing. **Michael J. Meaney**: funding acquisition, supervision. **Eric J. Nestler**: writing–review and editing, funding acquisition, supervision. **Stanley J. Watson Jr**.: supervision, funding acquisition, resources. **Huda Akil**: funding acquisition, supervision, resources.

## Conflicts of Interest

The authors declare no conflicts of interest. Several authors are members of the Pritzker Neuropsychiatric Disorders Research Consortium (M.H.H., H.A., and S.J.W.), which is supported by the Pritzker Neuropsychiatric Disorders Research Fund LLC. A shared intellectual property agreement exists between this philanthropic fund and the University of Michigan, Stanford University, the Weill Medical College of Cornell University, the University of California at Irvine, and the HudsonAlpha Institute for Biotechnology to encourage the development of appropriate findings for research and clinical applications.

## Peer Review

The peer review history for this article is available at https://publons.com/publon/10.1002/brb3.70608

## Supporting information



Supplementary Materials.

Supplementary Materials.

Supplementary Materials.

## Data Availability

The data that support the findings of this study are available in Gene Expression Omnibus at https://www.ncbi.nlm.nih.gov/geo/. These data were derived from the following resources available in the public domain: GSE89692: https://www.ncbi.nlm.nih.gov/geo/query/acc.cgi?acc=GSE89692, GSE116416: https://www.ncbi.nlm.nih.gov/geo/query/acc.cgi?acc=GSE116416, GSE14720: https://www.ncbi.nlm.nih.gov/geo/query/acc.cgi?acc=GSE14720, GSE153043: https://www.ncbi.nlm.nih.gov/geo/query/acc.cgi?acc=GSE153043, and GSE124387: https://www.ncbi.nlm.nih.gov/geo/query/acc.cgi?acc=GSE124387
