# Supplementary material for: A Meta‐Analysis of the Effects of Early Life Stress on the Prefrontal Cortex Transcriptome Reveals Long‐Term Downregulation of Myelin‐Related Gene Expression
Source: Brain Behav. 2025 Jun 17;15(6):e70608. doi: 10.1002/brb3.70608 (PMC12171234; doi:10.1002/brb3.70608)
Supplement: Supplementary file 3 — Supplementary Materials. [file BRB3-15-e70608-s001.docx]

**Supplementary Material for:**

**A meta-analysis of the effects of early life stress on the prefrontal cortex transcriptome reveals long-term down-regulation of myelin-related gene expression**

Toni Q. Duan^1^, Megan H. Hagenauer, Ph.D.^2*^, Elizabeth I. Flandreau, Ph.D.^3^, Anne Bader^1^, Duy Manh Nguyen^1^, Pamela M. Maras, Ph.D.^2^, Randriely Merscher S. De Lima, Ph.D.^4^, Trevonn Gyles^5^, Christabel Mclain^5^, Michael. J. Meaney, Ph.D., FRSC, C.Q., C.M.^6^, Eric J. Nestler M.D., Ph.D.^5^, Stanley J. Watson, Jr., M.D., Ph.D.^2^, Huda Akil, Ph.D.^2^

1. Grinnell College, Grinnell, IA USA;
2. Michigan Neuroscience Institute, University of Michigan, Ann Arbor, MI USA;
3. Psychology Dept., Grand Valley State University, Allendale, MI USA;
4. McGill University, Montreal, Montreal, QC, Canada;
5. Nash Family Department of Neuroscience and Friedman Brain Institute, Icahn School of Medicine at Mount Sinai, New York City, NY USA;
6. Douglas Hospital Research Centre, Verdun, QC, Canada;

Send correspondence to:

Megan Hagenauer, Ph.D.

Michigan Neuroscience Institute

University of Michigan

Ann Arbor, MI 48109

[hagenaue@umich.edu](mailto:hagenaue@umich.edu)

**Supplemental Table Legends**

***Table S1.*** ***The full meta-analysis results (11,889 genes, 11,885 stable meta-analysis estimates).*** *This .xlsx file includes two worksheets: 1) The worksheet “MetaAnalysisOutputByPval” provides the full meta-analysis results, with each row representing the results for one gene, and each column providing either gene annotation or meta-analysis statistical output. The results are ordered by p-value, so that the top rows in the worksheet are the genes with the smallest p-values. 2) The worksheet “ColumnDefinitions” provides the definitions for the variables present in each column in “MetaAnalysisOutputByPval”.*

***Table S2: The full fast Gene Set Enrichment Analysis (fGSEA) results (9322 gene sets).*** *This .xlsx file includes two worksheets: 1) The worksheet “fGSEA_Results” provides the full fGSEA results, with each row representing the results for one gene set, and each column providing the fGSEA statistical output. The results are ordered by p-value, so that the top rows in the worksheet are the gene sets with the smallest p-values. 2) The worksheet “ColumnDefinitions” provides the definitions for the variables present in each column in “fGSEA_Results”.*

**Supplementary Figures**


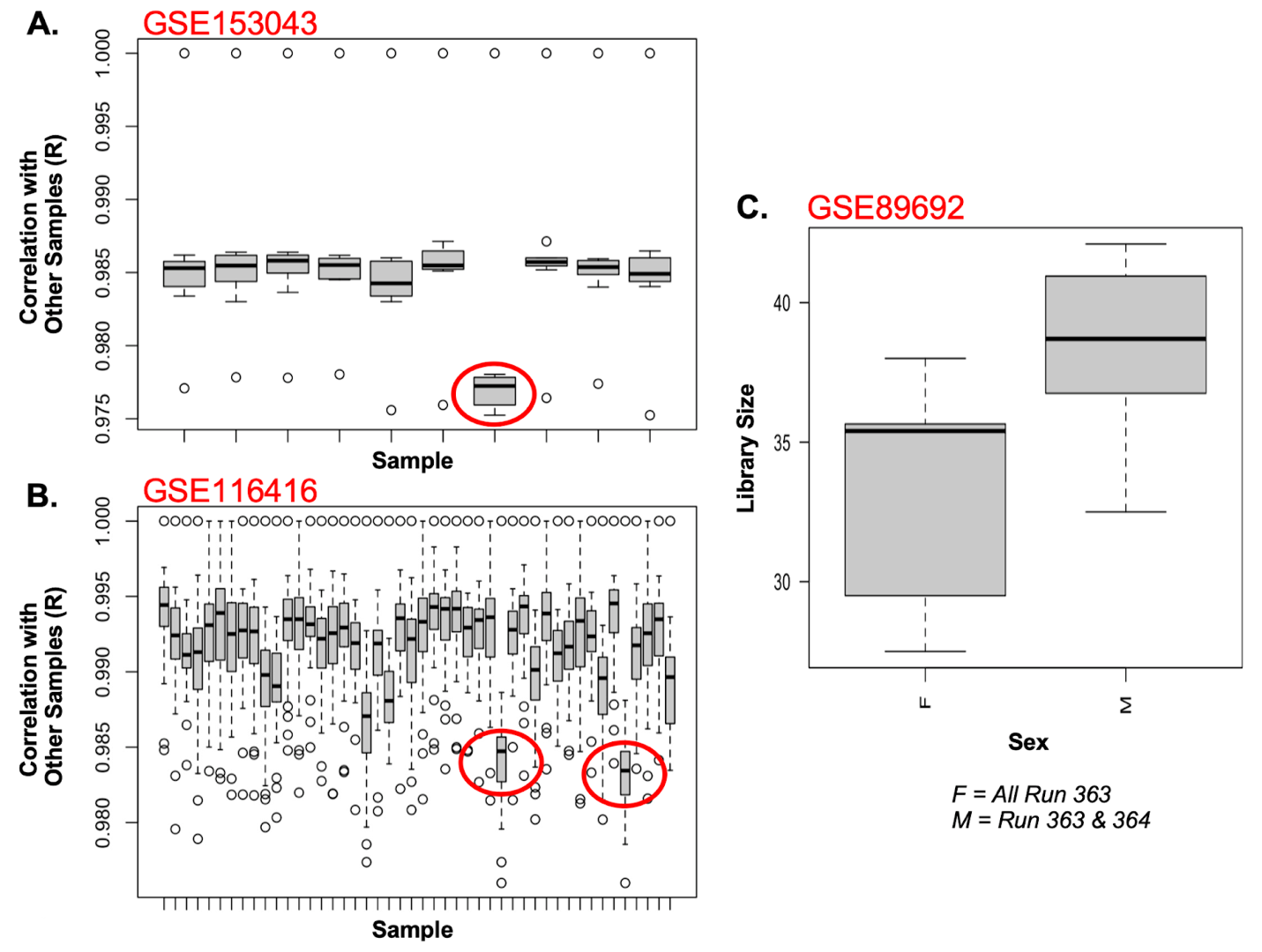


***Figure S1. Quality control decisions during preprocessing.*** ***A.*** *One outlier sample (circled red) was excluded from GSE153043 due to having full-genome gene expression patterns that had a notably low correlation with the full-genome gene expression patterns for all other samples in the dataset, suggesting an issue with technical processing.* ***B.*** *Two outlier samples (circled red) were excluded from GSE116416 due to having full-genome gene expression patterns that had a notably low correlation with the full-genome gene expression patterns for all other samples in the dataset, suggesting an issue with technical processing.* ***C.*** *The samples from each sex in GSE89692 appeared to be processed in separate batches, with female samples sequenced in a single run (363) that only partially overlapped with the male samples, and notably different library sizes associated with each sex, suggesting that there may have been sex-specific sample processing at some other critical technical step. This heterogeneity led us to include both sex and library size in our differential expression model.*

**A.**


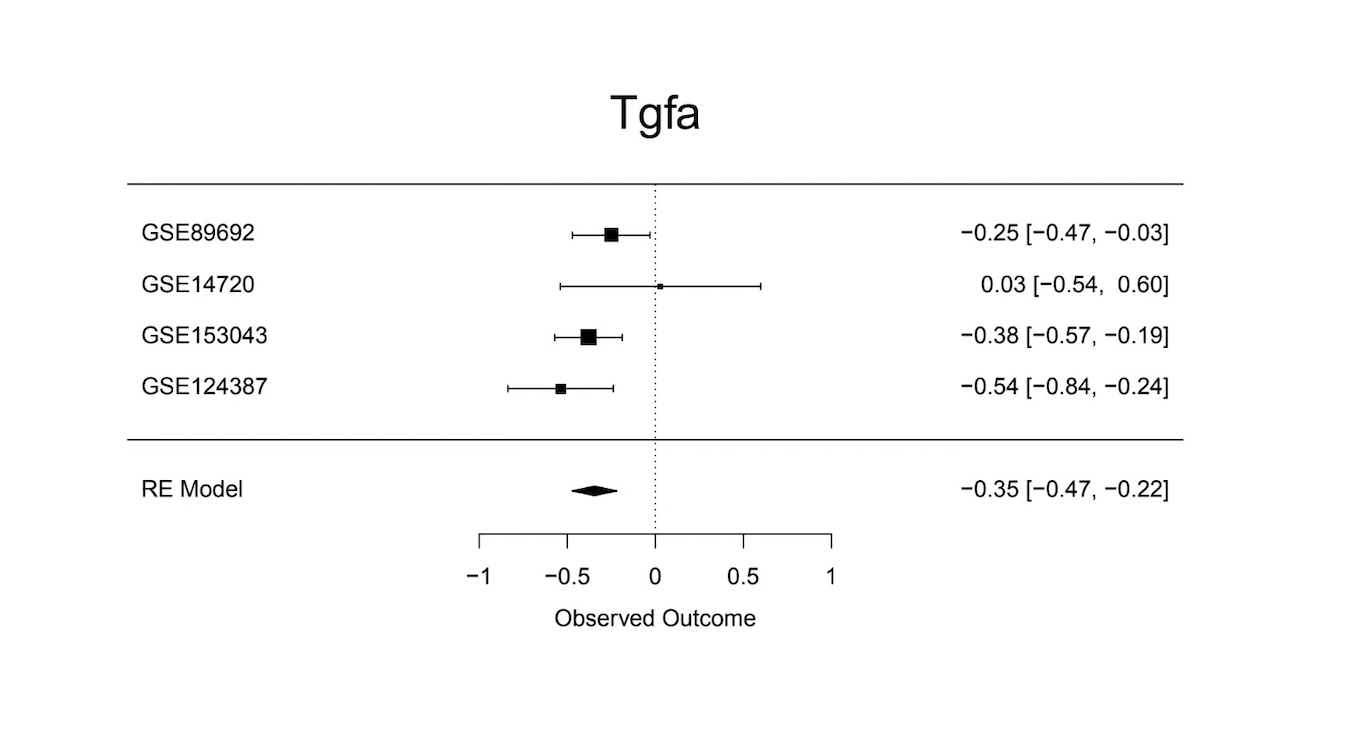


**B.**


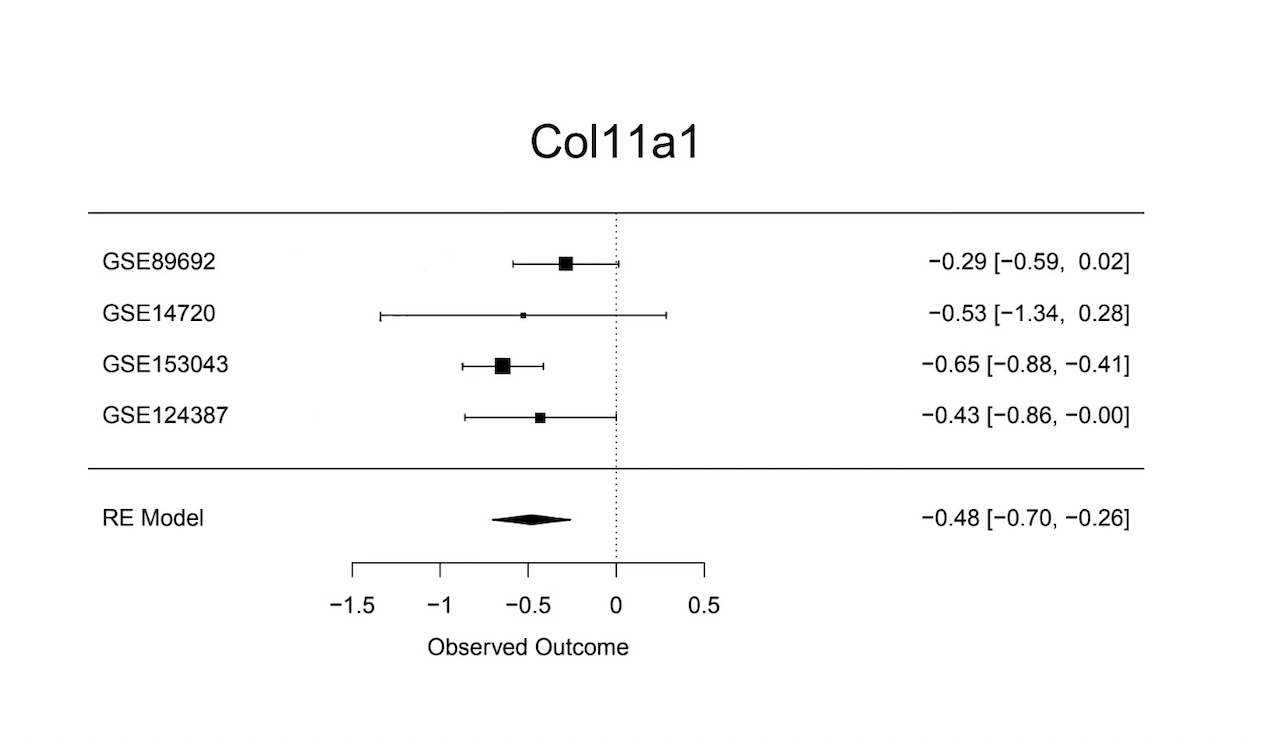


**C.**


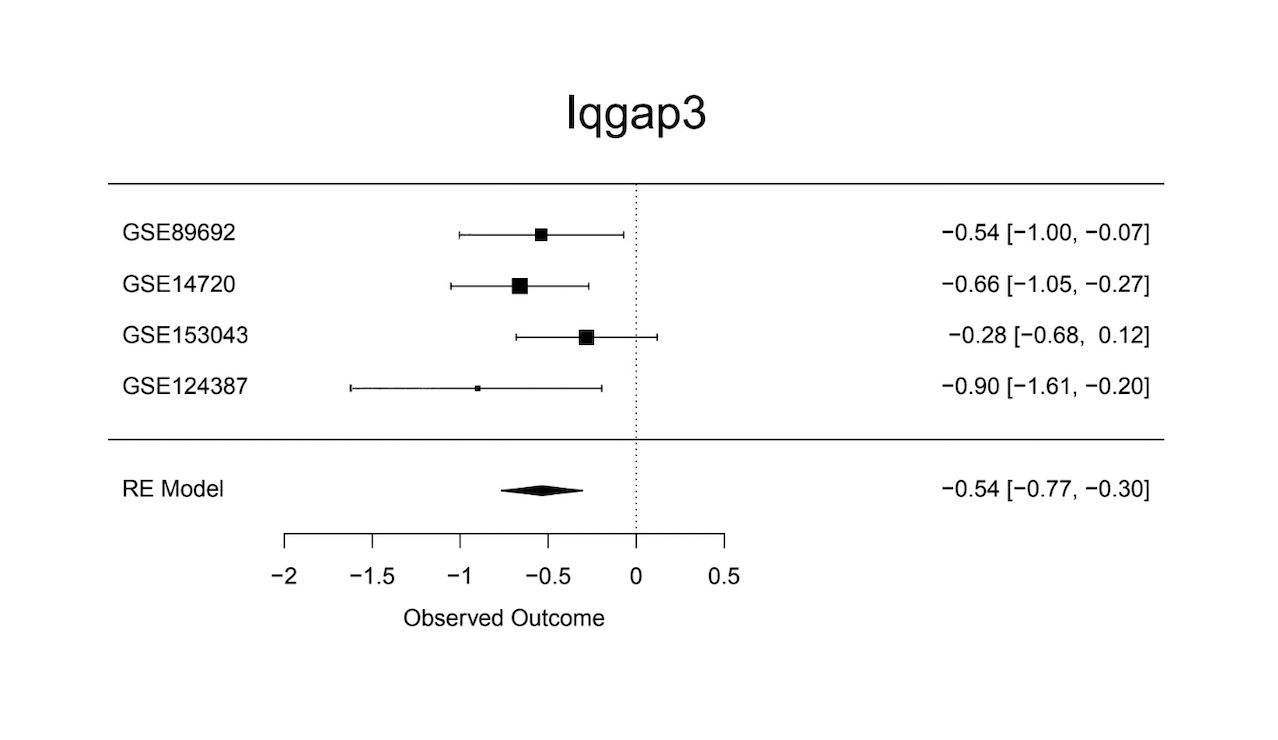


***Figure S2. Forest plots illustrating the differential expression following ELS. A-C.*** *Forest plots for the three differentially expressed genes from the meta-analysis (FDR<0.05) that weren’t illustrated in the main text. Rows illustrate ELS Log2FC (squares) with 95% confidence intervals (whiskers) for each of the datasets and the meta-analysis random effects model (“RE Model”). Forest plots allow for visual inspection of the consistency and magnitude of effects across the five studies.* ***A.*** *A forest plot showing the down-regulation of transforming growth factor alpha* (*Tgfa*) *in ELS models.* ***B.*** *A forest plot showing the down-regulation of collagen, type XI, alpha 1* (*Col11a1*) *in ELS models.* ***C.*** *A forest plot showing the down-regulation of IQ motif containing GTPase activating protein 3* (*Iqgap3*) *in early life stress models.*
